# Mitotic activity in non-Hodgkin's lymphoma. Relation to the Kiel classification and to prognosis.

**DOI:** 10.1038/bjc.1987.41

**Published:** 1987-02

**Authors:** M. Akerman, L. Brandt, A. Johnson, H. Olsson

## Abstract

At histopathological diagnosis of non-Hodgkin's lymphoma (NHL) the mean number of mitoses in 10 high power fields (X 40) was determined in thin sections (2 micron) and designated 'mitotic index' (MI). In 38 patients the thymidine labelling index (LI) of the lymphoma cells was also determined. There was a close correlation between MIs and LIs (r = 0.81, P less than 0.001) indicating that MI reflects the proliferative activity in NHL. Among 101 patients with NHL classified according to the Kiel nomenclature MIs were generally lower in lymphomas of low grade malignant type than in the high grade malignant lymphomas. The variation of MIs within morphological subgroups was especially pronounced in high grade lymphomas. Only 18 of 49 patients (37%) with MI greater than or equal to 2 have survived for 2 years in contrast to 37 of 52 patients (77%) with MI less than 2 (P = 0.001). For patients with histologically low grade lymphomas and MI greater than or equal to 2.0 the median survival was 23 months and for those with MI less than 2.0 58 months (P = 0.09). Patients with high grade lymphomas and MI greater than or equal to 2.0 had a median survival of 15 months compared to 57 months for those with MI less than 2.0 (P = 0.04). In a multivariate analysis of 50 patients with centroblastic-centrocytic (CB-CC) or centroblastic (CB) lymphomas the importance of different prognostic factors was analysed. Among the variables age, MI, growth pattern (follicular vs. diffuse), cell type (CB-CC vs. CB), clinical stage (I vs. II-IV), initial chemotherapy (active vs. less active) only age and MI gave significant prognostic information. It is concluded that the assessment of mitoses in NHL gives prognostic information in addition to histopathologic classification. The method is simple and the proliferative activity and histopathological diagnosis can be ascertained routinely on the same occasion.


*Br. J. Cancer* (1987), **55**, 219–223 © The Macmillan Press Ltd., 1987

### Mitotic activity in non-Hodgkin's lymphoma. Relation to the Kiel classification and to prognosis

M. Åkerman[1], L. Brandt[2], A. Johnson[2] & H. Olsson[2]

[1]*Department of Clinical Cytology and* [2]*Oncology, University Hospital of Lund, S-221 85, Lund, Sweden.*

**Summary** At histopathological diagnosis of non-Hodgkin's lymphoma (NHL) the mean number of mitoses in 10 high power fields (×40) was determined in thin sections (2 μm) and designated 'mitotic index' (MI). In 38 patients the thymidine labelling index (LI) of the lymphoma cells was also determined. There was a close correlation between MIs and LIs ($r=0.81$, $P<0.001$) indicating that MI reflects the proliferative activity in NHL. Among 101 patients with NHL classified according to the Kiel nomenclature MIs were generally lower in lymphomas of low grade malignant type than in the high grade malignant lymphomas. The variation of MIs within morphological subgroups was especially pronounced in high grade lymphomas. Only 18 of 49 patients (37%) with $MI \geqq 2$ have survived for 2 years in contrast to 37 of 52 patients (77%) with $MI < 2$ ($P=0.001$). For patients with histologically low grade lymphomas and $MI \geqq 2.0$ the median survival was 23 months and for those with $MI < 2.0$ 58 months ($P=0.09$). Patients with high grade lymphomas and $MI \geqq 2.0$ had a median survival of 15 months compared to 57 months for those with $MI < 2.0$ ($P=0.04$). In a multivariate analysis of 50 patients with centroblastic-centrocytic (CB-CC) or centroblastic (CB) lymphomas the importance of different prognostic factors was analysed. Among the variables age, MI, growth pattern (follicular *vs.* diffuse), cell type (CB-CC *vs.* CB), clinical stage (I *vs.* II-IV), initial chemotherapy (active *vs.* less active) only age and MI gave significant prognostic information.

It is concluded that the assessment of mitoses in NHL gives prognostic information in addition to histopathologic classification. The method is simple and the proliferative activity and histopathological diagnosis can be ascertained routinely on the same occasion.

In non-Hodgkin's lymphoma (NHL) histopathologic classification is of importance for evaluation of prognosis and choice of therapy. Among the various systems for classification used, the Kiel classification (Lennert, 1978) has proven valuable for these purposes (Cavallin-Ståhl *et al.*, 1981; Glimelius *et al.*, 1983; Brittinger *et al.*, 1984).

According to the results from several studies the determination of the proliferative activity of lymphoma cells gives considerable prognostic information in addition to morphologic classification. In these studies uptake of thymidine (Brandt *et al.*, 1981; Costa *et al.*, 1981; Kvaløy *et al.*, 1985) or the determination of cells in S phase by flow cytometry (Roos *et al.*, 1985) have been used to assess the proliferative activity of the lymphoma tissue.

In the present work the number of mitoses has been assessed in biopsy material from patients with NHL classified according to the Kiel classification. The prognostic importance of the method has been evaluated by analysis of survival of the patients.

### Materials and methods

### *Patients*

The material comprises 107 patients with NHL diagnosed in 1976–1983. In 6 patients the biopsy material was not considered optimal for an assessment of the number of mitoses. Thus 101 patients were included in the study. There were 54 men and 47 women aged 31–89 years (median 66 years). Twenty-three patients presented with a single involved site—11 with a nodal site and 12 with an extranodal lesion. The remaining 78 patients were in stage II-IV. All patients were followed for a minimum of two years or until death. Follow-up was performed in our hospital or in close collaboration with other hospitals in our region.

### *Histopathologic examination*

Five μm sections were cut from the biopsy specimens and stained with H & E and according to Gordon's and Sweet's method to demonstrate reticulin. From these sections the gross architecture of the lymphoma was evaluated, i.e. whether it was follicular, follicular and diffuse or diffuse. Thin, 2 μm sections were prepared and stained with H & E, May-Grünwald-Giemsa (MGG) and PAS according to McManus to evaluate the cellular composition of the lymphoma. In some cases imprint preparations from fresh lymphoma tissue were available and the classification was then based on a combined analysis of sections and imprints.

### *Determination of mitotic activity*

In the thin, 2 μm sections, stained with H & E the number of mitoses was recorded in 10 high power fields (×40). The total number of mitotic figures was divided by ten and the quotient was designated mitotic index (MI). In order to evaluate the reliability of MI, the number of mitoses was recorded on two occasions in 10 patients. On the second determination the pathologist was unaware of the outcome of the first assessment of MI. All determinations of MI were performed by one pathologist (M.Å).

### *Determination of labelling index*

Material from the lymphomas was obtained through aspiration. Cell suspensions were incubated with tritiated thymidine and the percentage labelled lymphoid cells (LI) was determined in autoradiography preparations as described previously (Brandt *et al.*, 1981).

### *Staging procedures*

Clinical staging was performed according to the Ann Arbor classification (Carbone *et al.*, 1971). The staging procedures have been described previously (Brandt *et al.*, 1981) and did not include staging laparotomy.

### *Treatment*

Histopathology, stage, age and performance status were the main factors used to determine treatment. Generally patients with localized disease, stage I-II, were treated with radio-

Correspondence: M. Åkerman.
Received 27 June 1986; and in revised form, 22 September 1986.

therapy and patients with disseminated disease, stage III-IV, received chemotherapy.

Patients with high grade malignant lymphomas were generally treated with CHOP (cyclophosphamide, doxorubicin, vincristine and prednisone), CHOP+methotrexate or MEV (methotrexate, cyclophosphamide and vincristine).

Patients with low grade malignant lymphomas were generally treated with either CVP (cyclophosphamide, vincristine and prednisone) or prednimustine, a chlorambucil ester of prednisolone. In those patients treated with CVP and/or prednimustine, who did not respond to treatment, the treatment was changed to CHOP or MEV.

Since the patients were diagnosed within a fairly long period of time, treatment has not been uniform. In the analysis of the impact of MI compared to other prognostic factors (see below) the initial therapeutic regimens were therefore dichotomized into intense (CHOP, CHOP+methotrexate and MEV) and less intense (CVP and prednimustine).

*Survival*

Survival was recorded from the date of diagnosis until death or date of last follow up.

The Kaplan-Meier method was used for the univariate estimation of survival time; differences in survival times were tested by generalized Wilcoxon statistics. For the follicle center cell lymphomas (FCC) of centroblastic-centrocytic (CB-CC) and centroblastic (CB) type a multivariate analysis was performed using Cox's proportional hazard analysis (Cox, 1972). By means of mathematical modelling, the impact of MI was evaluated in relation to known prognostic factors: age at diagnosis, growth pattern, lymphoma type, stage and treatment. The relative risk of dying was exemplified for the continuous variables age and MI. Each of the variables growth pattern, lymphoma type, stage and treatment was dichotomized into two categories and the relative risk was calculated for each factor. Two-sided $P$-values are given for the significant factors.

**Figure 1** Correlation between mitotic indices (MI) and labelling indices (LI) in 38 patients with non-Hodgkin's lymphoma.

### Results

*Repeated assays of MI*

The MIs recorded on two occasions in 10 patients are shown in Table I. In each patient the difference between the two MIs was small, on average 0.7.

**Table I** Results of repeated determinations of MI in 10 patients

| Assessment no. | | Mitotic indices 1 | Mitotic indices 2 | Difference |
|---|---|---|---|---|
| Patient no. | 1 | 0.5 | 0.3 | 0.2 |
| | 2 | 0.8 | 1.2 | 0.4 |
| | 3 | 0.8 | 1.2 | 0.4 |
| | 4 | 1.2 | 1.2 | 0.0 |
| | 5 | 2.5 | 2.9 | 0.4 |
| | 6 | 3.6 | 2.2 | 1.4 |
| | 7 | 7.2 | 8.1 | 0.9 |
| | 8 | 8.0 | 8.6 | 0.6 |
| | 9 | 8.3 | 9.6 | 1.3 |
| | 10 | 10.1 | 8.6 | 1.5 |

*Relation between MI and LI*

In 38 patients the MIs and LIs were determined (Figure 1). There was a significant correlation between the number of mitoses recorded and the number of cells in S phase as determined by LI (r=0.81, $P$<0.001).

*MI and morphology*

The MIs in various morphologic subgroups of NHL are shown in Figure 2. In low grade malignant lymphomas MIs were generally low and there was only a small variation of MI within the various morphologic groups. The centrocytic (CC) lymphomas constituted an exception with a marked heterogeneity. There were no obvious differences in MIs when comparing follicular *versus* diffuse growth pattern in CB-CC lymphomas.

In the high grade malignant lymphomas there was a considerable variation in MIs suggesting a great variation in proliferative activity.

*MI and survival*

In NHL a large proportion of patients surviving the first 2 years after diagnosis will be long term survivors (Leonard *et al.*, 1983). Forty-six patients have died within two years. The median MI for these patients was 2.8. For the 55 patients who have survived for more than two years the median MI was 1.3 (Figure 3). In order to further evaluate the prognostic impact of MI, the patient material was divided into two groups of about the same size: 49 patients with MI≧2.0 and 52 patients with MI<2.0. In the former group only 18 patients (37%) have survived for 2 years. Among patients with MI<2.0 37 patients (71%) have survived for more than 2 years ($\chi^2$=12.05, $P$=0.001).

*MI, morphology and survival*

Survival was significantly longer ($P$=0.003) in the low grade malignant lymphomas (median 57 months) compared to the high grade malignant lymphomas (median 15 months).

**Figure 2** Mitotic indices (MI) in different morphologic subgroups of non-Hodgkin's lymphoma according to the Kiel classification.
LC = lymphocytic, IC = immunocytic, CB-CC = centroblastic-centrocytic, CC = centrocytic, CB = centroblastic, IB = immunoblastic, LB = lymphoblastic, H = true histiocytic, unclass = unclassifiable.
(○) polymorphic immunocytoma, (▲) follicular or follicular + diffuse, (■) large cell.

**Figure 3** Mitotic index (MI) in relation to two years survival in patients with NHL. (○) dead intercurrently.

For patients with low grade malignant lymphomas and MI < 2 the median survival was 58 months and for those with MI ≥ 2 median survival was 23 months (Figure 4). The difference does not reach statistical significance ($P = 0.09$). For the patients with high grade malignant lymphomas and MI < 2 the median survival was 57 months compared to 15 months for those with MI ≥ 2 ($P = 0.04$, Figure 5).

A separate analysis of the prognostic impact of MI was performed in the patients with follicle center cell lymphomas (FCC) of the CB-CC and centroblastic (CB) subtypes. This group comprised 50 patients after exclusion of 3 patients who died from intercurrent disease. Age and MI were used as continuous variables. Growth pattern was categorized as either follicular or diffuse. Lymphomas with follicular and diffuse pattern were referred to the follicular group. Clinical stage was separated into two groups – stage I or stage II–IV. The lymphomas were dichotomized into CB-CC or CB. Initial treatment was divided into two groups – intense chemotherapy (CHOP, CHOP-M and MEV) or less intense (CVP and Prednimustine). The results are shown in Table II. Among the selected variables only age and MI gave significant prognostic information.

### Discussion

The present study was undertaken to investigate if the proliferative activity in NHL, estimated as the number of mitoses in histologic sections, may have prognostic implications. If the proliferative activity, measured as a mitotic count, is of prognostic value it was considered advantageous that the histopathologic diagnosis and MI can be determined on the same occasion.

Assessment of the number of mitoses calls for well fixed tissue without necrosis or crush artefacts. In our material these criteria were not fulfilled in 5% of the biopsies, although a diagnosis of NHL was possible to obtain. Even if optimal preparations are available the proposed method has

**Figure 4** Actuarial survival in 59 patients with low grade malignant lymphoma according to MI.

**Figure 5** Actuarial survival in 42 patients with high grade malignant lymphoma according to MI.

some pitfalls; the number of lymphoma cells may vary in different parts of a section. This is apparently not a serious drawback since the results of repeated determinations of MI were fairly constant. Another objection is that MI is not strictly related to the number of cells in the visual fields. In the large cell lymphomas, which constitute the high grade malignant group, the number of cells examined in 10 visual fields will naturally be lower than in the low grade malignant lymphomas. This might cause a relative underestimation of the MI in high grade malignant lymphomas.

**Table II** Prognostic factors in follicle center cell lymphomas. Cox's proportional hazard analysis. The relative risk is exemplified for each factor. *P* values are given for the significant factors

| *Factor* | *Relative risk* | *P value* |
|---|---|---|
| Age at diagnosis | | 0.05 |
| a difference of +20 years | 2.1 | |
| Mitotic index | | 0.03 |
| 2 *vs.* 1 | 1.6 | |
| 3 *vs.* 1 | 2.1 | |
| 4 *vs.* 1 | 2.7 | |
| 6 *vs.* 1 | 4.4 | |
| Growth pattern | | NS |
| diffuse *vs.* follicular | 1.4 | |
| Lymphoma type | | NS |
| CB *vs.* CB-CC | 1.2 | |
| Stage | | NS |
| II-IV *vs.* I | 1.4 | |
| Treatment | | NS |
| intense *vs.* less intense | 1.0 | |

In spite of these shortcomings the results indicate that this simple method might have clinical implications. The close correlation between MIs and LIs suggests that the determination of MI gives information on the proliferative activity of the lymphoma cells. Moreover, there was an association between MIs and the histopathological grading of malignancy, i.e. low grade malignant lymphomas had generally lower MIs than high grade ones.

Low MIs with only small differences among patients in the same subgroup were recorded in lymphocytic lymphomas and immunocytomas. There was a relatively wide range of variation of MIs in the CB-CC group and among the CC lymphomas there was considerable heterogeneity. The highest MI were recorded in lymphomas of high grade malignant type, and there was a striking variation in MI within the various subgroups. These findings are in good agreement with reports on the proliferative activity in NHL using other methods (Brandt *et al.*, 1981, Barlogie *et al.*, 1983, Costa *et al.*, 1981; Christensson, 1983; Hansen *et al.*, 1981; Roos *et al.*, 1985; Gerdes *et al.*, 1984).

The MI is obviously related to survival. About half of the patient material had a MI≧2.0. Only 37% of these patients have survived for more than two years whereas 71% of the patients with MI<2.0 are alive more than two years after diagnosis. Low grade malignant lymphomas with MI≧2.0 were associated with a shorter median survival time compared to low grade malignant lymphomas with MI<2.0. In high grade malignant lymphomas survival was significantly shorter for the patients with a MI≧2.0 than for those with a lower MI. Although the number of patients in each subgroup of low grade and high grade lymphomas was rather small, the results suggest that MI offers prognostic information in addition to the Kiel histopathological classification. It remains to be determined whether the assessment of MI is also valuable using other schemes of classification, e.g., the Working Formulation.

According to the Kiel nomenclature the follicle center cell (FCC) lymphomas constitute a large part of the NHL and the CB-CC (low grade) and CB (high grade) types are most common (Glimelius & Sundström, 1982; Brittinger *et al.*, 1984). The distinction between these two types of lymphomas may sometimes be uncertain. Moreover, it is sometimes difficult to ascertain whether the growth pattern is follicular, follicular and diffuse or entirely diffuse. Such distinctions might also be of prognostic importance. It was therefore considered worthwhile to further explore the prognostic value of MI in CB-CC and CB lymphomas. In a multivariate analysis age, growth pattern, lymphoma type, clinical stage, and initial treatment were prognostic factors tested for in comparison to MI. Age and MI turned out to be significant predictive factors and the others did not add further information. Thus the proliferative activity of CB-CC and CB lymphomas appears to be an independent prognostic variable.

With current treatment programs a low proliferative activity of lymphoma cells is compatible with long term survival (Brandt *et al.*, 1981; Costa *et al.*, 1981; Gronowitz *et al.*, 1983; Kvaløy *et al.*, 1985; Roos *et al.*, 1985). For patients with rapidly proliferating lymphomas, new therapeutic regimens are needed. A prompt and simple evaluation of the mitotic activity in histological preparations may help to select these patients for future therapeutic trials.

This work was supported by grants from the John and Augusta Persson Fund for Medical Scientific Research at the University of Lund, Sweden, and the Swedish Cancer Society (158–B86–19XB).

### References

BARLOGIE, B., RABER, M.N., SCHUMANN, J. & 6 others. (1983). Flow cytometry in clinical research. *Cancer Res.*, **43**, 3982.

BRANDT, L., OLSSON, H. & MONTI, M. (1981). Uptake of thymidine in lymphoma cells obtained through fine-needle aspiration biopsy. Relation to prognosis in non-Hodgkin's lymphoma. *Eur. J. Cancer Clin. Oncol.*, **11**, 1229.

BRITTINGER, G., BARTLES, H., COMMON, H. & 45 others. (1984). Clinical and prognostic relevance of the Kiel classification in non-Hodgkin's lymphomas. Results of a prospective multicenter study by the Kiel Lymphoma Study Group. *Hem. Oncol.*, **2**, 269.

CARBONE, P.P., KAPLAN, H.S., MUSSHOF, K., SMITHERS, D.W. & TUBIANA, M. (1971). Report on the committee on Hodgkin's disease staging classification. *Cancer Res.*, **31**, 1860.

CAVALLIN-STÅHL, E., LANDBERG, T., LINDBERG, L.G. & ÅKERMAN, M. (1981). A retrospective clinico-pathologic study of non-Hodgkin's lymphomas classified according to the nomenclatures of Lennert and Rappaport. *Acta Med. Scand.*, **209**, 407.

CHRISTENSSON, B. (1983). *Studies of non-Hodgkin's lymphoma: Immunological and flow cytometric DNA-analysis in relation to morphology and prognosis*. Thesis. Stockholm: Department of Pathology, Karolinska Institutet, Sweden.

COSTA, A., BONADONNA, G., VILLA, E., VALAGUSSA, P. & SILVESTRINI, R. (1981). Labeling index as a prognostic marker in non-Hodgkin's lymphomas. *J. Natl Cancer Inst.*, **66**, 1.

COX, D.R. (1972). Regression models and life tables. *J.R. Stat. Soc.*, **34**, 187.

GERDES, H., DALLENBACH, F., LENNERT, K., LEMKE, H. & STEIN, H. (1984). Growth fractions in malignant non-Hodgkin's lymphoma (NHL) as determined *in situ* with the monoclonal antibody Ki 67. *Hem. Oncol.*, **2**, 365.

GLIMELIUS, B. & SUNDSTRÖM, C. (1982). Morphologic classification of non-Hodgkin's lymphoma. Retrospective analysis using the Kiel classification. *Act. Rad. Oncol.*, **21**, 289.

GLIMELIUS, B., HAGBERG, H. & SUNDSTRÖM, C. (1983). Morphological classification of non-Hodgkin's malignant lymphoma. II. Comparison between Rappaport's classification and the Kiel classification. *Scand. J. Haematol.*, **30**, 13.

GRONOWITZ, S., HAGBERG, H., KÄLLANDER, C. & SIMONSSON, B. (1983). The use of serum deoxythymidine kinase as a prognostic marker, and in the monitoring of patients with non-Hodgkin's lymphoma. *Br. J. Cancer*, **47**, 487.

HANSEN,, H., KOZINER, B. & CLARKSON, B. (1981). Marker and kinetic studies in the non-Hodgkin's lymphomas. *Am. J. Med.*, **71**, 107.

KVALØY, S., MARTON, P.F., KAALHUS, O., HIE, J., FOSS-ABRAHAMSEN, A. & GODAL, T. (1985). $^{3}$H-thymidine uptake in B cell lymphomas. Relationship to treatment response and survival. *Scand. J. Haematol.*, **34**, 429.

LENNERT, K. (1978). In *Handbuch der speziellen patologischen Anatomie und Histologie, 1/3 Lymph nodes part B, Malignant Lymphomas other than Hodgkin's Disease*, p. 83, Springer-Verlag: Berlin, Heidelberg, New York.

LEONARD, R.C.F., CUZICK, J., MACLENNAN, I.C.M., VANHENGAN, R.I., MACKIE, P.H. & MCCORMICK, C.V., The Oxford Lymphoma group. (1983). Prognostic factors in non-Hodgkin's lymphoma: the importance of symptomatic stage as an adjunct to the Kiel histopathological classification. *Br. J. Cancer*, **47**, 91.

ROOS, G., DIGE, U., LENNER, P., LINDH, J. & JOHANSSON, H. (1985). Prognostic importance of DNA-analysis by flow cytometry in non-Hodgkin's lymphoma. *Hem. Oncol.*, **3**, 233.


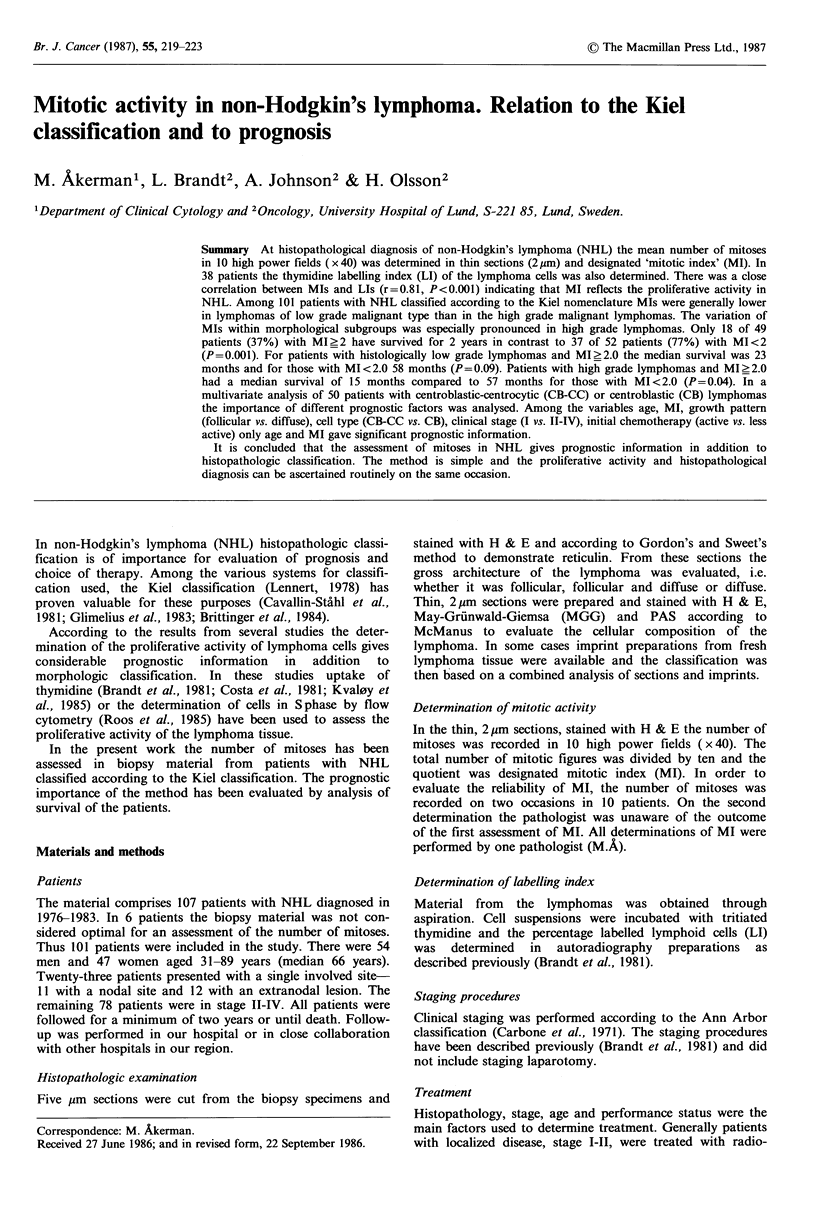

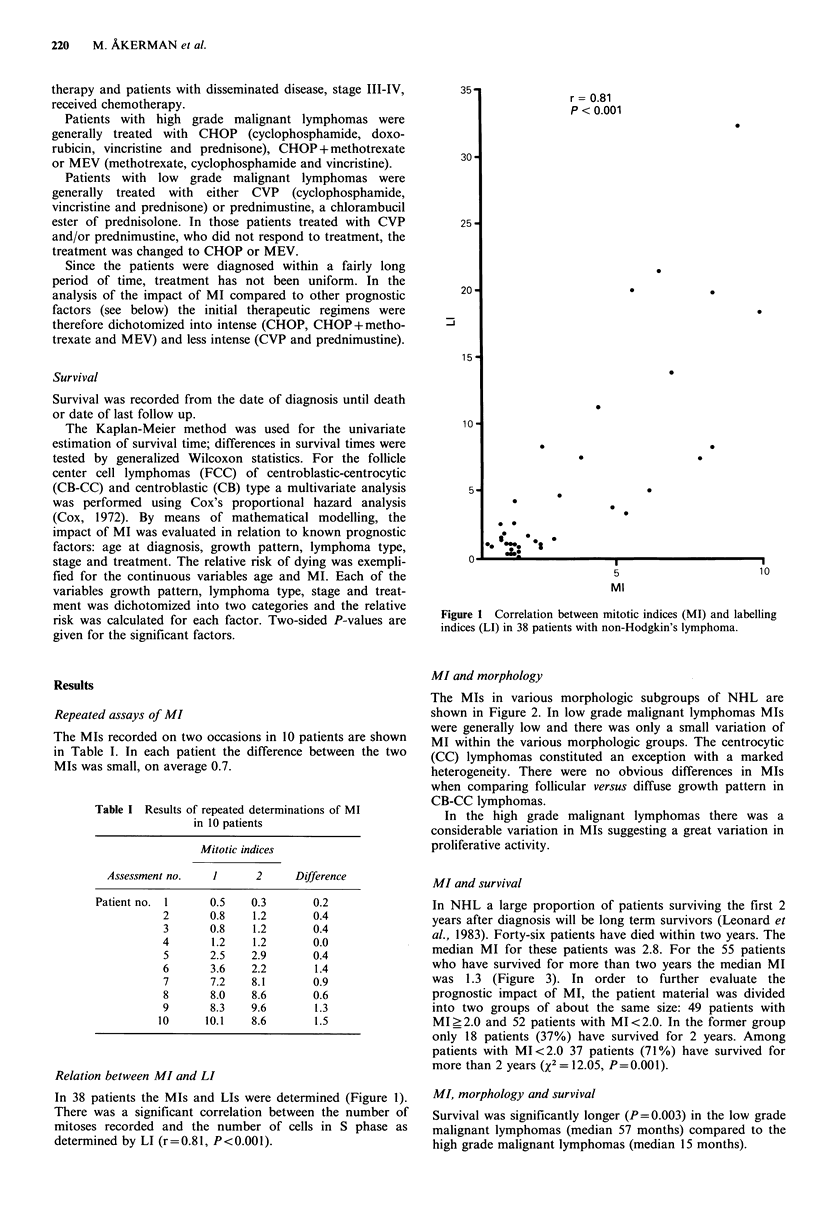

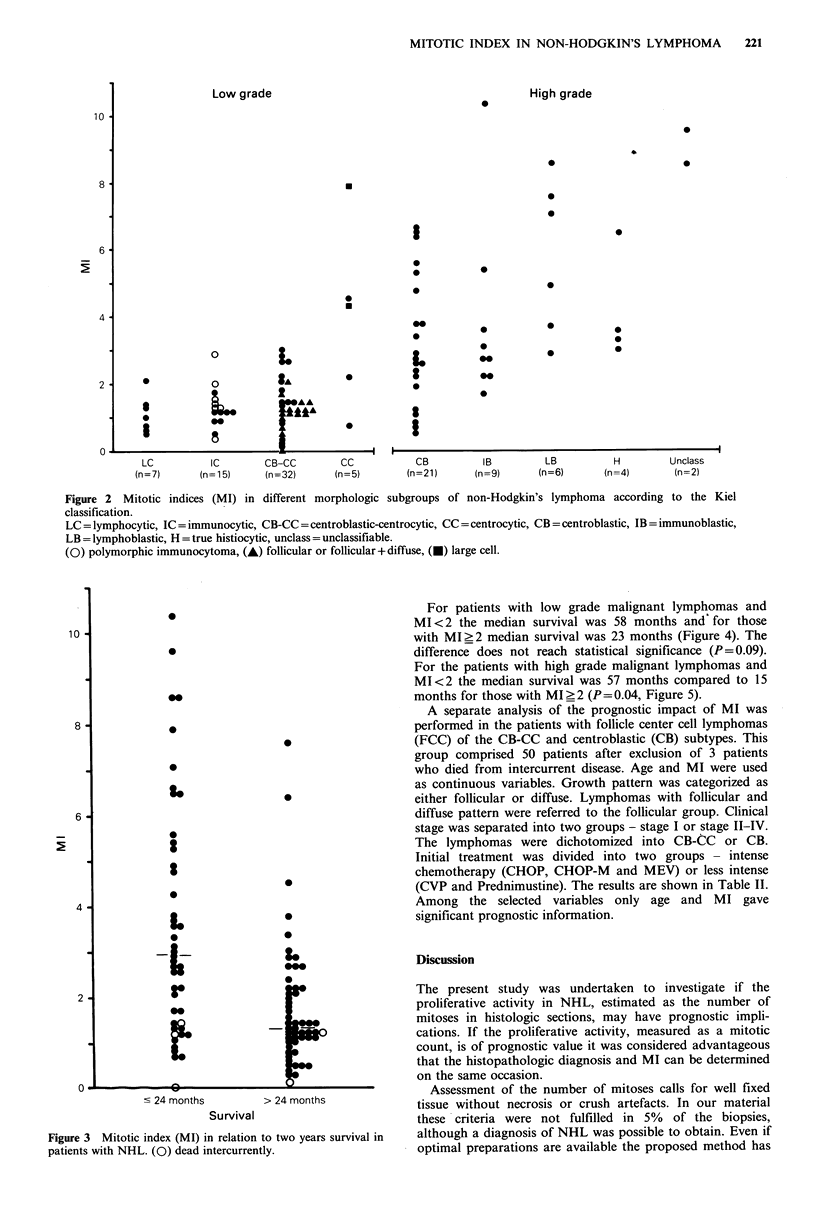

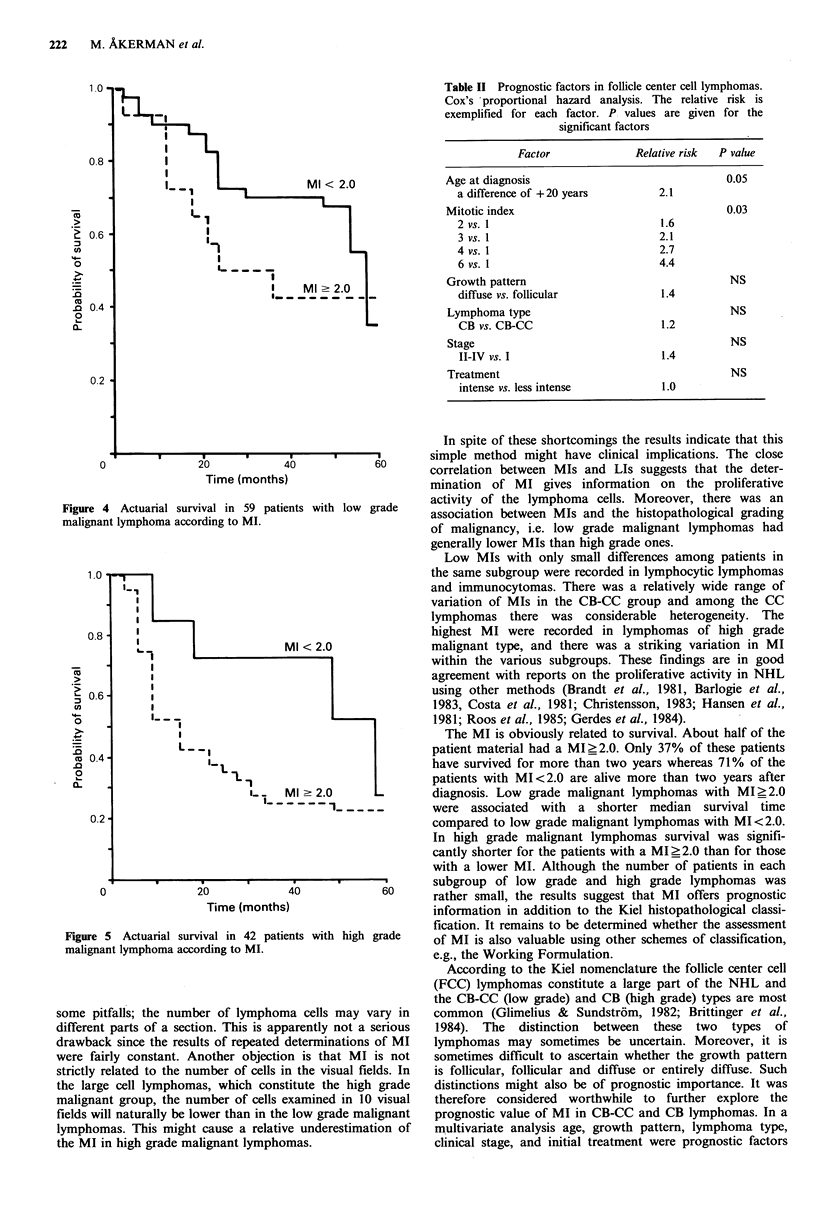

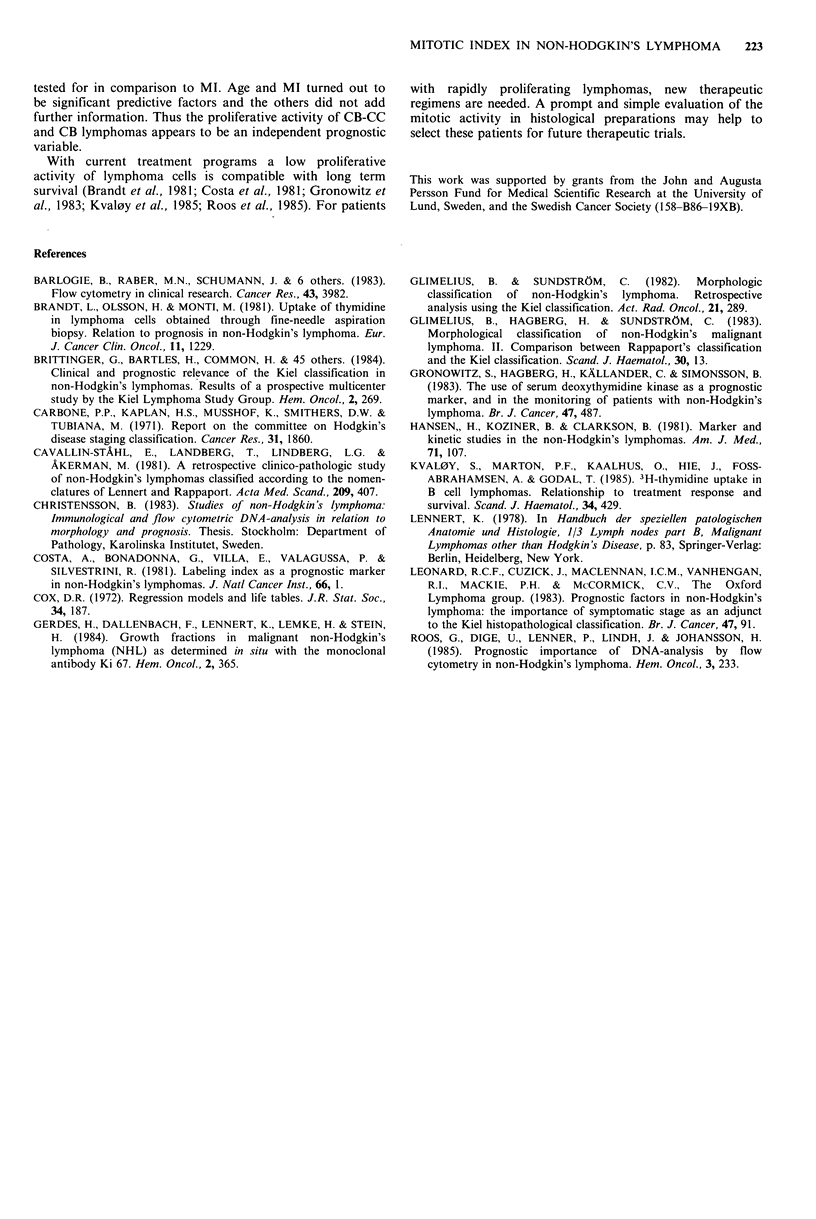


## References

[OCR_00649] Barlogie B., Raber M. N., Schumann J., Johnson T. S., Drewinko B., Swartzendruber D. E., Göhde W., Andreeff M., Freireich E. J. (1983). Flow cytometry in clinical cancer research.. Cancer Res.

[OCR_00653] Brandt L., Olsson H., Monti M. (1981). Uptake of thymidine in lymphoma cells obtained through fine-needle aspiration biopsy. Relation to prognosis in non-Hodgkin's lymphomas.. Eur J Cancer Clin Oncol.

[OCR_00659] Brittinger G., Bartels H., Common H., Dühmke E., Fülle H. H., Gunzer U., Gyenes T., Heinz R., König E., Meusers P. (1984). Clinical and prognostic relevance of the Kiel classification of non-Hodgkin lymphomas results of a prospective multicenter study by the Kiel Lymphoma Study Group.. Hematol Oncol.

[OCR_00664] Carbone P. P., Kaplan H. S., Musshoff K., Smithers D. W., Tubiana M. (1971). Report of the Committee on Hodgkin's Disease Staging Classification.. Cancer Res.

[OCR_00669] Cavallin-Ståhl E., Landberg T., Lindberg L. G., Akerman M. (1981). A retrospective clinico-pathologic study of non-Hodgkin's lymphomas classified according to the nomenclatures of Lennert and of Rappaport.. Acta Med Scand.

[OCR_00681] Costa A., Bonadonna G., Villa E., Valagussa P., Silvestrini R. (1981). Labeling index as a prognostic marker in non-Hodgkin's lymphomas.. J Natl Cancer Inst.

[OCR_00690] Gerdes J., Dallenbach F., Lennert K., Lemke H., Stein H. (1984). Growth fractions in malignant non-Hodgkin's lymphomas (NHL) as determined in situ with the monoclonal antibody Ki-67.. Hematol Oncol.

[OCR_00701] Glimelius B., Hagberg H., Sundström C. (1983). Morphological classification of non-Hodgkin malignant lymphoma. II. Comparison between Rappaport's classification and the Kiel classification.. Scand J Haematol.

[OCR_00696] Glimelius B., Sundström C. (1982). Morphologic classification of non-Hodgkin's lymphoma. I. Retrospective analysis using the Kiel classification.. Acta Radiol Oncol.

[OCR_00707] Gronowitz J. S., Hagberg H., Källander C. F., Simonsson B. (1983). The use of serum deoxythymidine kinase as a prognostic marker, and in the monitoring of patients with non-Hodgkin's lymphoma.. Br J Cancer.

[OCR_00713] Hansen H., Koziner B., Clarkson B. (1981). Marker and kinetic studies in the non-Hodgkin's lymphomas.. Am J Med.

[OCR_00720] Kvaløy S., Marton P. F., Kaalhus O., Høie J., Foss-Abrahamsen A., Godal T. (1985). 3H-thymidine uptake in B cell lymphomas--relationship to treatment response and survival.. Scand J Haematol.

[OCR_00737] Roos G., Dige U., Lenner P., Lindh J., Johansson H. (1985). Prognostic significance of DNA-analysis by flow cytometry in non-Hodgkin's lymphoma.. Hematol Oncol.

